# Novel therapeutic strategy for obesity through the gut microbiota-brain axis: A review article

**DOI:** 10.22088/cjim.15.2.215

**Published:** 2024

**Authors:** Romina Kardan, Jaber Hemmati, Mohsen Nazari, Amjad Ahmadi, Babak Asghari, Mehdi Azizi, Mansoor Khaledi, Mohammad Reza Arabestani

**Affiliations:** 1 Department of Neuroscience and Addiction Studies, School of Advanced Technologies in Medicine, Tehran University of Medical Sciences, Tehran, Iran; 2 Student Research Committee, Hamadan University of Medical Sciences, Hamadan, Iran; 3 Department of Microbiology, School of Medicine, Hamadan University of Medical Sciences, Hamadan, Iran; 4 Department of Tissue Engineering and Biomaterials, School of Advanced Medical Sciences and Technologies, Hamadan University of Medical Sciences, Hamadan, Iran; 5 Department of Microbiology and Immunology, School of Medicine, Shahrekord University of Medical Sciences, Shahrekord, Iran; 6 Nutrition Health Research Center, Hamadan University of Medical Sciences, Hamadan, Iran; # These authors contributed equally in this article.

**Keywords:** Gut–brain axis, Obesity, Gut microbiota, Probiotic, Prebiotic, Fecal Microbiota transplantation

## Abstract

**Background:** The interaction between commensal bacteria and the host is essential for health and the gut microbiota-brain axis plays a vital role in this regard. Obesity as a medical problem not only affect the health of the individuals, but also the economic and social aspects of communities. The presence of any dysbiosis in the composition of the gut microbiota disrupts in the gut microbiota-brain axis, which in turn leads to an increase in appetite and then obesity. Because common treatments for obesity have several drawbacks, the use of microbiota-based therapy in addition to treatment and prevention of obesity can have other numerous benefits for the individual. In this review, we intend to investigate the relationship between obesity and the gut microbiota-brain axis as well as novel treatment strategies based on this axis with an emphasis on gut microbiota.

## Introduction

The gut microbiota can be considered one of the important components of the body, having more than 100 times the human genome, weighing approximately 1–2 kg and consisting of trillions of living microorganisms are among the reasons for this claim (1, 2). Altering the composition of gut microbiota and conversion of rich/diverse into pathogenic/ abnormal bacteria, called gut dysbiosis, is associated with several disorders (e.g., obesity, type 2 diabetes mellitus) (3, 4). Impairment of gut homeostasis and nutrient metabolism, reduction of beneficial microbial metabolites and colonization of pathogens can be the most main causes of dysbiosis (5). Obesity results from an imbalance between energy intake and consumption. In addition, obesity is a multifactorial condition and one of the main health problems in societies that increases the mortality rate and economic and social damage (6, 7).The outbreak of obesity, which is determined by body mass index (BMI) ≥30 kg / m2, has increased intensely in recent decades and according to the World Health Organization in 2022, overweight and obesity affect almost 60% of adults and nearly one in three children in the European region (8). These factors have led to the widespread research of obesity, although there are limited treatment options today (9). 

The gut has a complex and bidirectional relationship with the central nervous system (CNS), which is termed as the gut microbiota–brain axis and plays a crucial role in homeostasis and, consequently obesity. Many studies in this field have been performed on humans and animals, the results of which indicate a close and crosstalk communication between the gut microbiota and the CNS (10, 11). In addition, the essential role of the gut microbiota in the development and evolution of the brain has been proven (12-14).

Also, disruptions in gut microbiota–brain axis have been implicated in the pathogenesis of several neurological disorders such as; Parkinson's disease, Autism spectrum disorder, Alzheimer's disease, Multiple Sclerosis (MS), Amyotrophic Lateral Sclerosis (ALS), Huntington’s disease, etc. (15). In this review, we discuss the role of the gut microbiota-brain axis in obesity and novel therapeutic strategies for this disorder.

**2. Gut Microbiota and Obesity**

Most gut microbiota of healthy adults is composed of *Bacteroidetes* and *Firmicutes* (2). In individuals with obesity compared with fit controls, it has been observed that the Bacteroidetes / Firmicutes ratio is decreased (16). Moreover, a comparison of the composition of the gut microbiota of obese or fit twins revealed, the proportion of *Firmicutes* did not differ significantly and *Actinobacteria* increased, while the diversity of bacteria and proportion of *Bacteroidetes* decreased (17). There has been evidence that the composition of the gut microbiota can be affected by weight loss (18, 19). Moreover, it has been proposed that the gut microbiota in obese people may harvest energy more efficiently, which may result in more fat accumulation than lean individuals (20). Research indicates that colonizing the microbiota of mice that were conventionally raised with germ-free mice significantly increased body fat even though the mice consumed less food (21). Furthermore, the gut microbiota donors from obese mice resulted in more weight gain than compared with lean donors (20, 22). Nevertheless, the findings are not consistent and comprehensive and cannot be generalized to humans (23).Obesity is associated with chronic low-grade inflammation, which is a risk factor for comorbid diseases such as diabetes, cardiovascular disease, and cancer (24, 25). The exact mechanisms of this inflammatory state are still unclear, and the gut microbiota is one of the factors involved in this condition (26). Altered microbiota composition in obese people increases the permeability of the intestinal barrier, leading to the passage of compounds caused by bacterial lysis such as endotoxin (LPS) into the bloodstream. Increased LPS levels in the blood stimulate pro-inflammatory cytokines and lead to inflammation in the nervous system (27, 28). Also, in the study on obese adults compared to the control group, after receiving a high-fat meal, serum LPS levels were much higher (29), indicating the role of the gut microbiota in the inflammatory state of obesity (30, 31).

**3. The Microbiota and Gut-Brain Axis**

The gut–brain axis plays an essential role in modulating the body's energy. The gut transmits nutritional signals through various pathways consisting of the vagus nerve, the enteric nervous system (ENS) as well as enteroendocrine cells (EECs) (Figure 1) (31). The various microbial metabolites that we discuss below, can regulate these signals.

Because EECs are located throughout the intestinal epithelium cells, these cells can interact with nutrients. Neurotransmitters and hormones such as serotonin (5-hydroxytryptamine), cholecystokinin (CCK), peptide YY (PYY), ghrelin and glucagon-like peptide 1 (GLP-1), transmit signals and activate these cells (30). These endocrine hormones can affect gut motility, feeding (through vagus neurons or ENS), and essential metabolism (secretion of insulin, gastric acid, and bile acids) (32, 33). Further, metabolites produced by the gut microbiota including short-chain fatty acids (SCFAs), can modulate the release of these neurotransmitters and hormones (34). 

Sensory signals of the intestine are transmitted to CNS via the vagus nerve, and this nerve plays a key role in signaling between the gut and the brain. This nerve has the longest neurons in the human body (35) and 80% of the fibers of these neurons are afferent and 20% of them are efferent (36). The anti-inflammatory properties of this nerve have been proven, and they are involved in gut motility and feeding (37). Alterations in the gastrointestinal tract, microbial-derived metabolites, and neurotransmitters produced by EECs stimulate or inhibit the vagus nerve (38). In this case study, it was found that vagotomy in mice affects body weight, indicating the role of the vagus nerve in metabolism and food intake (39). Several studies have also suggested an association between dysbiosis in obesity and vagus nerve signaling (40, 41).

The ENS is an extensive neural network through the gastrointestinal tract and controls the secretion/ assimilation of metabolites and gastrointestinal motility. This system can transmit information to the CNS both directly and through the vagus nerve (42). ENS function is not impaired by the loss of the vagus nerve and is a reason for the vagus nerve to be independent (43). The gut microbiota may signal the ENS through bacterial metabolites and neurotransmitters produced by EECs. Researches in germ-free mice found the role of gut microbiota in the maturation and function of the ENS (44, 45). 

Furthermore, in the gut microbiota-brain axis, microbiota-derived metabolites such as, dopamine, serotonin, gamma-aminobutyric acid (GABA) and SCFAs (lactate, butyrate, propionate, etc.) improve the performance of this axis and thus enhance communication between the two organs. The SCFAs have an important role in energy homeostasis by binding to G protein-coupled receptors (GPR) (46). In the animal study found that SCFAs-mediated activation of GPR induced glucose metabolism in both liver and muscle and inhibited fat storage in adipose tissues in mice (47). Moreover, these metabolites prevent fat accumulation in two direct and indirect pathways: In the direct pathway, SCFAs enter the CNS by stimulating the vagus nerve, which ultimately suppresses food intake (48). In the indirect pathway, SCFAs affect satiety by secreting PYY and GLP- and interacting with EECs (49, 50). In the study on the role of SCFAs in obesity, it was found that SCFAs-fed mice did not gain weight induced high-fat-diet (51). Studies have also shown the beneficial function of SCFAs in metabolism and energy consumption in humans (52-54). The role of acetate, butyrate, and propionate in obesity, which are among SCAFs, has been studied in several types of research. The study found that acetate induces anorexia in the hypothalamus and modulates satiety bypassing the BBB (55).

 In addition, a study demonstrated that acetate increases thermogenesis and energy consumption in obese mice (56). Also, butyrate is an essential source of energy for colonocytes (57) and the study of mice has shown that butyrate and propionate cause intestinal gluconeogenesis (58). 

**Figure 2:**
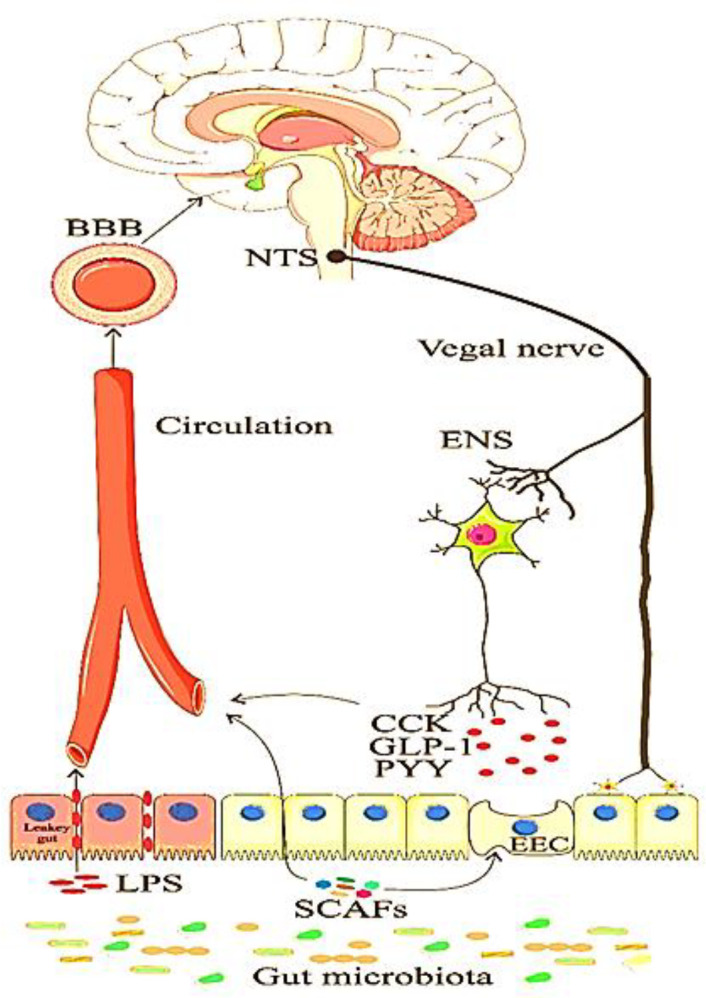
The microbiota and gut-brain axis. The communication between the brain and the gut takes place through different pathways as well as the metabolites produced by the gut microbiota, directly and indirectly, effect this axis. Microbial-derived metabolisms enter the bloodstream and penetrate the BBB and also effect on the EECs. EECs produce neurotransmitters that act on the axis through ENS, Signaling through the vagus nerve. Due to obesity and dysbiosis, increased intestinal permeability (Leaky Gut) and LPS cross the intestinal barrier and cause endotoxemia. SCFAs, short-chain fatty acids; LPS, lipopolysaccharide; BBB, blood-brain barrier; EEC, enteroendocrine cell; ENS, enteric nervous system; CCK, cholecystokinin; PYY, peptide YY; GLP-1, glucagon-like peptide; NTS, nucleus of tractus solitarius

**4. Gut Microbiota and Feeding Behavior**

Food consumption is vital for survival, and signals in times of hunger and satiety trigger food-motivating behaviors that are regulated by pathways that interact closely with the gut microbiota (59).

**4.1. Homeostatic Pathway**

The homeostatic pathway regulates energy metabolism and energy balance by peptides released from the EECs (e.g. CCK, PYY,GLP-1), hormones (e.g. leptin and ghrelin) and microbiota-derived metabolites (e.g. SCFAs) (Figure 2) (60). The arcuate nucleus (ARC) is a part of the hypothalamus that plays a leading role in controlling food intake by receiving messages transmitted through the vagus nerve and activating the nucleus of tractus solitarius (NTS) (61, 62). Two main neuronal groups are involved in feeding and modulating energy. The first group are orexigenic GABAergic neurons and the second group are the anorexigenic glutamatergic neurons. GABAergic neurons increase food intake by releasing aguti-related protein (AgRP) and neuropeptide Y (NPY) and reduce energy consumption (63). The glutamatergic neurons reduced food consumption by releasing pro-pionemocortin (POMC) that express alpha-melanocyte-stimulating hormone (α-MSH). This hormone stimulates the melanocortin satiety pathway through the melanocortin-4-receptor (MC4R). Also, glutamatergic neurons play a role in the expression of cocaine- and amphetamine-regulated transcript (CART) (63). 

The hormones leptin and ghrelin play a significant role in the regulation of hemostatic pathway. Leptin is an anorexic hormone that indicates a long-term energy balance and is produced by white adipose tissue. The low plasma levels of leptin indicate starvation signals (64). Leptin, after passing through the BBB, binds to its receptors in the ARC, suppressing NPY/AgRP neurons and stimulating POMC/CART neurons, which leads to the induction of satiety in the body (65). 

High levels of leptin do not always lead to a reduction in food intake, also a study has shown that serum leptin levels in obese adults are higher than in fit controls, indicating resistance to the effects of leptin in these people (similar to insulin resistance in type 2 diabetes) (66). It was also observed that weight loss did not occur with the treatment of exogenous leptin in obesity, indicating resistance to the effects of leptin (67). 

The strongest hypothesis in the mechanism of resistance of leptin is the disruption of hypothalamus signaling (68). Ghrelin is also an orexigenic hormone that increases stomach mobility and secretes gastric acid (69) and is mainly secreted by EECs in the stomach (70). Plasma level of this hormone rises before meals and this amount decreases after mealtimes and during digestion (71). Also, ghrelin, after crossing BBB, directly activates NPY/AgRP neurons and inhibits of POMC/CART by releasing GABA (72, 73). In addition to stimulating food intake, ghrelin is involved in glucose metabolism, reward behavior, and sense of taste (69). 

The gut microbiota is associated with the concentration of hormones that regulate food intake behaviors. *Bifidobacterium* spp. and *Lactobacillus *spp. are among the most important genera of the gut microbial composition that have a positive correlation with serum leptin levels and a negative correlation with serum ghrelin levels (74). It was shown that germ-free mice lost more weight than the control group after leptin administration, indicating that these mice were more sensitive to leptin (75). Also, treatment of patients infected with *Helicobacter pylori*, in addition to increasing the ratio of Bacteroidetes/Firmicutes reduced the plasma level of ghrelin, which indicates the effect of the gut microbiota composition on ghrelin concentration (76). Furthermore, a study has proven SCFAs and different bacteria, including *Bifidobacterium* and *Lactobacillus* genera suppress ghrelin signaling (through the growth hormone receptor1a) and motivate leptin production (through the activation of GPR41) (77). Administration of a probiotic bacterium, such as *Lactobacillus rhamnosus* GG (LGG) increases the sensitivity to exogenous leptin and reduced the proportion of Bacteriaidets/ Firmicutes and Proteobacteria in fecal microbiota in mice with dietary-induced obesity (78). 

Anorexia peptides of CCK, GLP-1 and PYY which are secreted by EEC, are also another important factor in regulating the hemostatic pathway. CCK is the first hormone in human that reduce food intake and provoke satiety through activation of the vagal neurons (79). This hormone stimulates the digestion of fat and protein through the release of digestive enzymes and bile from the pancreas and gallbladder (80). A recent study has proven that dysbiosis reduces satiety-associated CCK as a result of decreased vagus nerve signaling at the NTS (81). GLP-1 peptide increases and decreases insulin and glucagon secretion, respectively, and modulates blood glucose levels and also one of the therapeutic targets for type 2 diabetes mellitus are GLP-1RAs (GLP-1 receptor agonists) (82). In addition GLP-1RAs activate and suppresses POMC/CART and POMC/CART neurons, respectively, which induces satiety and thus weight loss (83). PYY also plays an important role in food intake and increasing the plasma level of this peptide after meal inhibits NPY/AgRP and subsequently suppresses POMC neurons, which leads to anorexia (84).

SCFAs are among the microbiota-derived metabolites that bind to EECs and alter the secretion of intestinal hormones (79). Acetate is one of the major SCFAs produced by the gut microbiota, which directly reduces appetite by affecting the hypothalamus (55), and increased dysbiosis-induced acetate activates the parasympathetic nervous system, increased insulin and ghrelin secretion and as a result becomes obesity (85). Numerous studies have shown that the production of SCFAs and the secretion of intestinal hormones increase with the fermentation of non-digestible carbohydrates by the gut microbiota (55). In addition, it has been proven that the expression of intestinal satiety peptides has decreased in germ-free mice (86). Also, propionate has anti-inflammatory properties and promotes the secretion of leptin in human adipose tissue (87).

**Figure 3:**
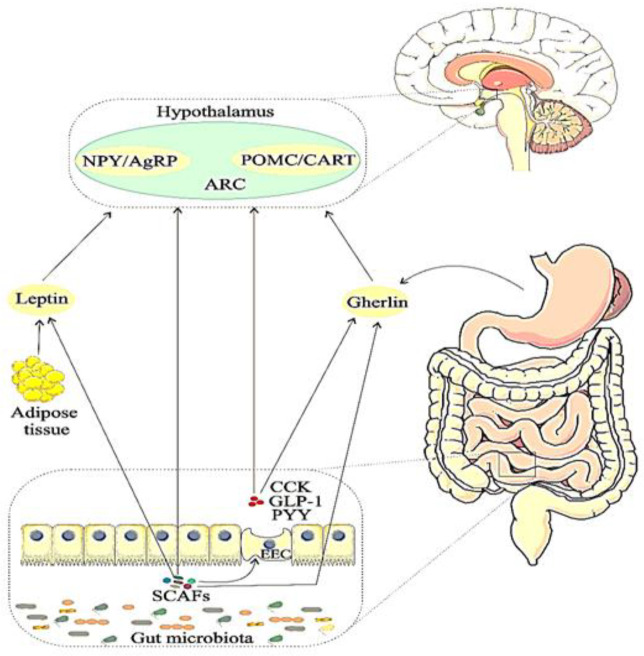
The gut microbiota affects feeding behavior through the homeostatic pathway. This pathway includes the processing of signals from leptin and microbial-derived peptides, CCK, GLP-1, PYY, and ghrelin. Changes in the composition of the gut microbiota (dysbiosis) can affect the dopaminergic pathway. ARC, arcuate nucleus; POMC, pro-opiomelanocortin; CART, cocaine- and amphetamine-regulated transcript; NPY, neuropeptide Y; AgRP, agouti-related protein; SCFAs, short-chain fatty acids; PYY, peptide YY; CCK, cholecystokinin; GLP-1, glucagon-like peptide-1; EEC, enteroendocrine cell

**4.2 Dopaminergic pathway**

The dopaminergic mesolimbic system mediated brain reward signaling. This system, which is concentrated in the striatum, is closely related to the hemostatic pathway and causes obesity (84). Dopamine is one of the important neurotransmitters in neurological processes that perform important functions such as cognition, learning and motivation (88).

 Also, dopamine exerts its action by binding to specific membrane receptors. Moreover, the dopamine function depends on the its release site (89). Large amounts of circulating dopamine is produced through the interaction between the gut microbiota and EECs in the gastrointestinal tract, which reduces intestinal motility and regulates mucosal blood flow (90). Dopamine is released after food intake and causes a feeling of hedonic reward, which stimulates feeding behavior (91). In some cases studies, it has been reported that in several dopaminergic brain disorders such as anxiety, depression, and Parkinson’s disease, the composition of gut microbiota has altered and the inflammatory conditions caused by dysbiosis are associated with these disorders (92). 

It has been proven that some bacterial species play an important role in appetite and food intake by affecting reward signals (93). In addition, a human study has shown that propionate reduces the reward response to a high-energy meal suggesting, that SCFAs play a role in regulating the dopaminergic pathway (88). 

**4.3 Another neurotransmitter**

In addition to dopamine, GABA and serotonin are neurotransmitters produced by the gut microbiota that affect appetite in the brain and peripherally (94-96). GABA is an inhibitory neurotransmitter that controls the hypothalamic control of food intake (96) and GABA-producing bacteria improve host metabolic condition. Also bacteria microbiota including *Lactobacillus* and *Bifidobacterium* increase GABA levels (97). The study also found that in obese individuals, GABA plasma levels increased after fecal microbiota transplantation (FMT) from lean donors (98).

Serotonin is one of the essential neurotransmitters involved in regulating satiety, secretion and glucose metabolism (99). Serotonin suppresses food intake by inhibiting NPY/AgRP and activation of POMC neurons (100). EECs are one of the main production sites of peripheral serotonin and there is also evidence to regulate the peripheral concentration of serotonin through the gut microbiota (101). But there is still not enough information about the effect of the gut microbiota on central serotonin.

**5. Therapeutic strategy for obesity**

Given the key role of the gut microbiota in the modulating appetite, energy homeostasis and host metabolism, it is not unexpected that the microbiota is currently a target for the prevention and treatment of metabolic disorders such as obesity. However, more studies are needed before gut microbiota-based therapy is used as a therapeutic tool to suppress appetite and food intake and restore metabolic imbalances in obesity and other metabolic disorders (102, 103) . We aim to discuss novel microbiota-based therapy for the prevention and treatment of obesity and in this section probiotics, prebiotics, and FMT mechanisms of action and their effects on obesity will be explained.

**5.1 Probiotics**

Probiotics are living microorganisms that affect health if taken in sufficient amounts and the most common bacteria used as probiotics are *Bifidobacterium* and *Lactobacillus *genera (104). 

Novel sequencing techniques such as Metagenomics confirm the significant role of the gut microbiota, both locally and systematically. The researchers suggested that a better understanding of the probiotics’ mechanism of action on host metabolism and homeostasis and their effect on gut microbiota-brain axis modulation, reduces host sensitivity to obesity and other metabolic diseases and also uses it as a treatment option (105). 

Extensive research has been conducted on the relationship between probiotics and weight changes in animals and humans, and probiotics are considered as one of the appropriate treatment strategies for obesity (106). The development of the gut microbiota and proper balance between pathogens and the microorganisms in this composition are among the effects of probiotics, that play this role through 3 mechanisms: antimicrobial activity, immunomodulation and intestinal barrier support (105, 107). 

**5.1.1 Antimicrobial Activity**

Reduction of luminal pH, inhibiting of bacterial adherence and secretion of antimicrobials substances are among the activities of probiotics to prevent pathogens colonization in of the gut (108). In addition, hydrogen sulfide-production and the decrease of oxidation / reduction potential are conditions caused by probiotics and play an important role in combating adverse microorganism (109). In addition to reducing colonic pH and inhibiting the colonization of pH-sensitive pathogens, SCAFs (produced by the fermentation of carbohydrates) have also been shown to increase the production of butyrate precursors (110, 111). In this regard, the study showed that *Bifidobacteria* spp. and *Firmicutes* spp. were less sensitive to lumen acidic conditions compared to *Bacteroides* spp. (109). 

Also, have been proven using *Lactobacillus* alters the composition of gut microbiota, especially pathogens, by decreasing the pH of the lumen resulting in the production of lactic acid (112). 

Antimicrobial peptides (AMPs) such as bacteriocins, are including products of probiotics that prevent the overgrowth of pathogens. For example, several species of *Lactobacillus species *have been described as precursors of different bacteriocins (113, 114). Defensins are also among the AMPs and cationic proteins and are considered as one of the innate components of immunity in various organisms. Several studies have shown that various genera of bacteria, such as *Pediococcus* and *Lactobacillus*, induce defensins production (115).

**5.1.2 Immunomodulation**

The gut microbiota affects the innate and acquired immune systems by controlling epithelial cells, dendritic cells (DCs), macrophages and lymphocytes through various mechanisms.

Epithelial cells can produce signals and cytokines to distinguish commensal and pathogenic bacteria from each other (116). One study described that the reduced function of the epithelial barrier as a result of pro-inflammatory cytokines is repaired by the *Lactobacillus rhamnosus* GG. In addition, in acute and chronic inflammation that where is in the inner layer of the colon, probiotics such as *B. infantis*, *L. casei*, *L. plantarum* and *L. brevis* are considered as treatment candidates (117, 118).

The DCs interact with gut bacteria, especially those that have access to the M-cells in the Peyer's patches (119, 120). DCs also produce IL-10 and TGF-β from regulatory T cells and IgA from B cells (121). Several studies have examined the effect of probiotics on DCs, and in a study found that DCs induce IL-10 from intestinal tissue and also have an inhibitory effect on T helper-1 cells (122). In addition to DCs, tissue macrophages present antigens to memory T cells (123). Bacteria such as *Lactobacillus casei* modulates the production of IL-10 and IL-12 through macrophages, indicating the role of probiotic immunomodulation (124). The effect of probiotics on lymphocytes has been investigated in clinical studies and it has been shown that *L. reuteri* modulates inflammation conditions in the intestine by increasing FOXP3 induction and thus the development of T regulatory cells (Figure 3) (125).

**5.1.3 Intestinal Barrier Support**

The structure of the intestinal lumen is composed of a monolayer epithelium between the mucosal membrane and the lamina propria. The mucus on the epithelial layer is secreted by the goblet cells and separates the gut microbiota from the epithelial cells (126). Increased permeability of the intestinal barrier causes abnormal and excessive cross of bacteria and their products through the epithelial layer, which causes chronic low-grade inflammation and, as mentioned earlier, is contributed to obesity (127). Moreover, the disruption of the intestinal barrier is associated with diseases such as type 1 diabetes (128), celiac (129), enteric infection (130) and type 2 diabetes (129).

Probiotics have been proven that play an important role in improving intestinal barrier function by modulating the phosphorylation of tight junctional and cytoskeletal proteins as well as provoking mucus secretion, and can also prevent dysbiosis. In the case study, it was shown that *Lactobacillus acidophilus* and *Bifidobacterium infantis* restore the intestinal barrier function by regulating occludin and claudin-1 (tight junctional protein) (127). In addition, *Lactobacillus acidophilus *and *S. thermophilus *can preserve and strengthen of intestinal permeability the epithelial cell line invaded by Enteroinvasive *E. coli* (EIEC) (131). Further, several strains of *Lactobacilli*, also induced mucin gene expression in HT29 and Caco-2 cell lines (both of are derived from intestinal cancer) and inhibited adherence and invasion of the pathogenic *E. coli* (132).

**5.2 Prebiotics**

Prebiotics are compounds that increase the growth of beneficial bacteria in the gut and have a positive effect on physiology metabolic function. Prebiotics enhance human health by improving the genetic capacity of the gut microbial and increasing the production of beneficial metabolites (133). For example, studies have shown that prebiotics increases the responses of anorexia peptides (GLP-1, PYY) and through the hemostatic pathway can play an anti-obesity role (134, 135). Prebiotic fibers are the main source of SCFAs and play an effective role in the production of butyrate. In addition to its anti-inflammatory properties, butyrate induces the secretion of mucosal IgA from B cells and the development of T regulatory cells (136, 137). 

Prebiotics increase the growth of *Lactobacillus* spp. and *Bifidobacterium* spp. to other bacteria in the gut microbial populations which are called the bifidogenic effect. In this regard, infants feeding with high-fiber supplements increase in the ratio of *Bifidobacterium* spp. compared to the prebiotic-free supplement group (138). Moreover, the study has shown that high-fiber diets play an important role in weight loss by increasing tight junction and decreasing pro-inflammatory cytokines and endotoxemia (139). 

In addition, prebiotics are involved in the decrease of pro-inflammatory cytokine production, reducing the luminal pH, increasing the mineral absorption (e.g., Ca^2+^, Mg2+, Fe^2+^) and inhibiting the growth of pathogenic bacteria (105).

**Figure 3:**
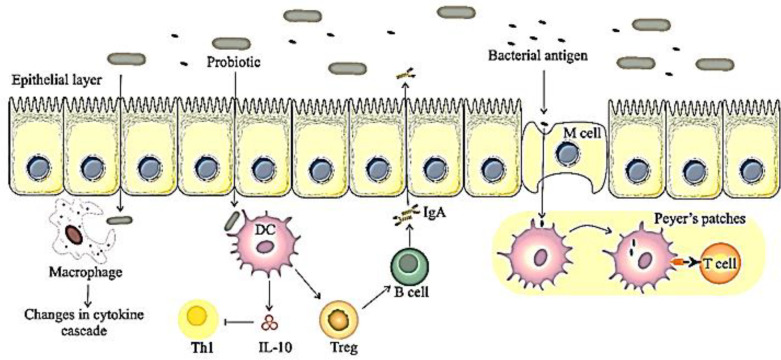
Immunomodulation mechanisms of probiotics. Probiotics interact with epithelial cells, DCs, macrophages, B cells, Treg and Peyer's patches. DCs, dendritic cells; B cell, B lymphocytes; Treg, regulatory T cells; T cell, T lymphocytes

**5.3 Fecal Microbiota Transplantation (FMT)**

FMT is a useful solution to change the composition and function of the microbiota gut by already infusing the feces of healthy individuals to the recipient (140). FMT by increasing the diversity of gut microbiota and their metabolites such as SCFAs can also have positive physiological effects on the recipient (141).

One of the conditions that increases the chance of survival of transmitted bacteria is the preceding presence of these strains in the recipient (142).

 The high efficacy (more than 90%) of FMT for the treatment of *Clostridium difficile* infections (143), as well as its low side effects (mild fever and diarrhea) (144), make it a suitable candidate treatment for ulcerative colitis, chronic constipation, irritable bowel syndrome and other diseases of intestinal dysbiosis (140). In the study on FMT from a person donor with a normal BMI to an obese recipient with type 2 diabetes, it was shown that insulin sensitivity increased. Therefore, in addition to obesity, type 2 diabetes mellitus is another disease that FMT can have the potential to treat, but many studies need to be done to make a definite conclusion (141).

Gut -brain axis is a strong and vital link between the brain and the gut, that plays a prominent role in increased appetite and consequently obesity. Modulating the gut microbiota is achieved through mechanisms such as probiotics, prebiotics, and FMT that are new and appropriate therapeutic strategies for obesity. Also, probiotic can develop the gut microbiota and consequently, treatment of obesity through pathways including; antimicrobial activity, immunomodulation and intestinal barrier support. Further, the use of these strategies and clarifying their mechanisms and impacts on human health requires further investigation.
